# Making the most of food aid to help prevent child and maternal deaths

**DOI:** 10.9745/GHSP-D-13-00084

**Published:** 2013-08-14

**Authors:** Bruce Cogill

## Abstract

Advances in child nutrition over the last several decades are creating momentum for a programmatic push to reduce undernutrition worldwide. The contribution of food aid may be small, but, nonetheless, U.S. food aid policy should be revamped to benefit more effectively and more efficiently the children and mothers in need.

The recent series on nutrition in *The Lancet,*^1^ together with a paper by Tappis et al.^2^ in this issue of GHSP, provide an opportunity to reflect on the great progress in nutrition that has been achieved in the past few decades. Child survival and nutrition statistics tell a compelling story of both the accomplishments and also the challenges. Despite alarming increases in the numbers of overweight or obese children, undernutrition among children continues to decrease in much of the world. According to the *Lancet* series, the estimated number of children under 5 years who are stunted has steadily dropped, from 253 million in 1990, to 178 million in 2005, and to 164.8 million in 2011.^1^ These worldwide decreases are led by historically unprecedented improvements in Latin America and parts of Asia, especially China and Southeast Asia. The decline in numbers globally has occurred while global population has grown by 31% since 1990. Unfortunately, however, the numbers of stunted children in sub-Saharan Africa continue to increase.

Specific nutrition and health interventions probably have played only a supportive role in the decline in global numbers. The combination of greater economic opportunities, education, gender and social equity, political engagement, and social protection; improved infrastructure; and public nutrition and health programs has saved lives and changed the planet. This has been the case in countries such as Brazil, China, and Thailand, and even in developed countries over the last 100 years.

Consensus is now emerging that concerted programmatic actions can successfully address undernutrition and, increasingly, overnutrition and that we know what the priority actions are. The global development agenda is seized with the belief that “…*we know what to do; let's just do it.*” This has a familiar ring to it, and, as the *Lancet* series attests, along with the communication buzz, there is enthusiasm at the United Nations, in nongovernmental organizations (NGOs), and at many levels of governments in high- and low-income countries^3^ to take action to prevent or even eliminate the undernutrition that affects billions of people.

## KEY ROLE OF NUTRITION

The goal of ending preventable child and maternal deaths has taken on unprecedented priority and attention on the global health stage.^4^ The *Lancet* series estimates that about 45% of child deaths and almost the same proportion of maternal deaths have underlying nutritional causes, with suboptimal breastfeeding alone accounting for 11% of child deaths.^1^ And the *Lancet* series, along with the Scaling Up Nutrition agenda (http://www.scalingupnutrition.org) and many others, have identified a range of higher-impact interventions for infants and children, women of reproductive age, pregnant women, and, significantly, adolescents (Table).^5^ These interventions have been selected based on studies that show a measurable and scalable impact. They are the starting point for determining what, either as a single effort or, preferably, in combination, is appropriate for the setting in which you are working.

**TABLE.** The 10 Key Interventions for Women and Children^a^

**Optimum Maternal Nutrition During Pregnancy**
1. Periconceptual folic acid supplementation or fortification
2. Maternal balanced energy protein supplementation
3. Maternal multiple micronutrient supplementation in pregnancy
4. Maternal calcium supplementation
**Infant and Young Child Feeding**
5. Promotion of breastfeeding
6. Appropriate complementary feeding
**Micronutrient Supplementation in Children at Risk (ages 6–59 months)**
7. Preventive zinc supplementation
8. Vitamin A supplementation
**Management of Acute Malnutrition**
9. Management of severe acute malnutrition
10. Management of moderate acute malnutrition

a Based on their ability to save lives and be most cost-effective, assuming 90% coverage in the 34 high-burden nutrition countries. Source: Adapted from Bhutta et al.^5^

## ROLE OF FOOD AID

When we consider what can be done and how it can be supported, development assistance or aid is often the main focus of debate. Levels, targets, accountability, governance, transparency, and messaging are all part of the discussion.

Food assistance is an important part of the U.S. aid budget, contributing to nutrition, health, and agricultural programs in many recipient countries. Assistance for young children and their families is now the mainstay of food aid. Globally, direct food transfers have a somewhat minor role in addressing undernutrition overall, but they have a role with certain vulnerable populations that is essential to ending preventable child and maternal deaths. Food assistance is one resource considered only briefly in the *Lancet* series, and yet it is often placed by politicians and advocacy groups next to development assistance as worthy and needed. Cash transfers and in-kind assistance is covered in the series, but except for the case of emergencies, food aid receives cursory attention.

Food aid used in non-emergency settings carries with it decades of debate as to whether it is inefficient and even damaging to the recovery of local agriculture-based economies. The reasons for its persistence are many, reflecting more the domestic policy environment than the value or effectiveness of the food aid per se. As a continuing resource for improved nutrition and food security, food aid needs to work better and differently. In their paper in this issue, Tappis et al.^2^ offer a case study of some of the challenges in a U.S. food aid program. The authors document the logistic and management experiences that faced NGOs implementing a development, or non-emergency, food aid program in South Sudan both before and after independence from Sudan on July 9, 2011.

By recounting the flow of food aid and the missteps that occurred, the authors argue for more flexibility in the rules that govern the procurement, movement, and programming of U.S. food aid. They also join many others in suggesting that more local procurement of commodities in the country or region will improve efficiency, improve dietary diversity and quality, and support local agriculture. The local procurement of foods, the authors argue, would diminish concerns over the likely presence of genetically modified (GM) foods, such as soy and corn, in the U.S. food aid basket. Setting aside the issue of GM foods' benefits or otherwise, the description of the food aid program in an unstable region does illustrate some of the many limitations and challenges that come with the food aid program, which is part of the massive U.S. Farm Bill.

## STATUS OF FOOD AID REFORM

Every 5 years or so, the U.S. Congress renegotiates the Farm Bill, which is the authorizing legislation for the sprawling and symbiotic food and agricultural ecosystem that drives domestic and global food policies and programs. As I write this editorial, the current round of reauthorization is in legislative limbo, and stakeholders; lobbyists from agriculture, food manufacturing, and transportation industries; and implementers such as the World Food Programme and NGOs are making vigorous efforts to influence the specifics in the bill being crafted by the powerful U.S. House and Senate Agricultural Committees.

Understanding the specifics of this US$955 billion annual behemoth, especially as it relates to health- and nutrition-related support, requires patience and motivation. The bill typically funds the domestic U.S. Food Stamps program (now called Supplemental Nutrition Assistance Program, or SNAP) and food aid used in emergency and non-emergency programs in many parts of the world. The most recent House version of the Farm Bill has removed the provision for the SNAP program to facilitate passage of the remaining bill swollen with subsidies for domestic agricultural programs. Understanding the dynamics of the evolving Farm Bill will help us understand some of the challenges in the South Sudan example.

The United States continues to supply the global relief and development effort while insisting on mostly U.S.-grown, U.S.-processed, U.S.-packaged, and U.S.-shipped food. In both emergency and non-emergency situations, health clinics and other sites are still distributing food in the form of U.S. commodities, such as fortified corn-soya blend, vegetable oil, and legumes. Without question, emergency food will need to come from afar in times of clearly defined acute food shortages, as is sometimes the case after an extended drought, other weather-related event, or civil unrest. But, for a sack of a blended cereal to travel 7,000 miles and be given to a caregiver constitutes an inefficient use of the resources of many people, including those of the health worker and the caregiver.

## WHAT'S NEEDED FOR FOOD AID REFORM

The food aid program and the Farm Bill have been the constant subject of analysis and recommendations for reform by economists,^6^ policy analysts, journalists,^7^ and others.^8^ Proposals by the U.S. Agency for International Development (USAID) and others^9^ include:

- Allowing greater flexibility in what is funded and how it is funded to provide flexibility in programming and more rapid responses—a move away from a tied, commodities-only approach
- Pairing in-kind food aid procurements from the United States with the use of cash-based interventions such as food vouchers and local and regional procurement from developing countries
- Ending the costly and inefficient process of monetization, or selling U.S. food aid commodities in affected and neighboring countries to raise cash for local programs

The United States is still committed to supplying food, but the almost 60-year-old program is no longer a surplus commodity program helping to distribute food that would otherwise be lost. It is one of the many entrenched federally funded entitlement programs managed out of government agencies in Washington, DC, led by a range of interest groups and lobbyists.

Nevertheless, the reform proposals from USAID and others clearly would be a major step in the right direction. The need is to examine closely the use of U.S. commodities in food aid, especially for the health and nutrition non-emergency or development programs. There is less need to revamp the provision of life-saving food and health care to vulnerable populations affected by natural and man-made disasters.

With the renewed emphasis on nutrition, its champions challenge us to move forward with a wide array of actions to reduce over- and undernutrition. These advocates for nutrition action from governments, UN agencies, academies, civil society, food manufacturers, and others therefore should champion an effort to refocus food aid to benefit most those it claims to help. Food aid is only a small part of the overall effort to end preventable child and maternal deaths; nonetheless, U.S. food aid policy needs to reflect the good intentions of its providers, the American people, and also an economic and humanitarian rationale.
